# Characterization and Evolutionary Analysis of Type E4 Bovine Enterovirus From Clinical Cattle With Diarrhea in Inner Mongolia, China

**DOI:** 10.1155/tbed/2973871

**Published:** 2026-09-18

**Authors:** Lin Hou, Jia-Lei Zhang, Ya-Xing Ban, Qi An, Zhi-Chao Li, Yi-Yang Zou, Zi-Yuan Li, Qiang Du, Xuan An, Shuai Liu, Zi-Tong Wang, Zhi-Dan Zhang, Chun-Xia Chai, Ni-Ni-Gen Xi, Wei-Guang Zhou

**Affiliations:** ^1^ College of Veterinary Medicine, Inner Mongolia Agricultural University, Hohhot, China, imau.edu.cn; ^2^ Key Laboratory of Clinical Diagnosis and Treatment Technology of Animal Diseases, Ministry of Agriculture, Hohhot, China, moa.gov.cn; ^3^ Animal Disease Prevention and Control Center of Ulanqab, Ulanqab, China

**Keywords:** bovine enterovirus, diarrhea, evolutionary analysis, pathogenicity, viral load

## Abstract

Bovine enterovirus (BEV) is associated with respiratory, intestinal, and reproductive disorders in cattle, but its evolutionary characteristics and pathogenic potential remain insufficiently understood. In this study, a BEV strain was successfully isolated from a diarrheic fecal sample collected from an 8‐month‐old Holstein calf and was designated BEV NM2407. The virus replicated stably in Madin‐Darby bovine kidney (MDBK) cells, reaching a titer of 10^9.3^ TCID_50_/mL. Its complete genome was 7414 nt in length, and phylogenetic analysis classified the isolate as EV‐E4. Recombination analysis identified two putative breakpoints at 1950 and 5850 nt, and the resulting genomic regions showed the highest similarity to EV‐C‐ and EV‐D‐related reference lineages. VP1 analysis revealed a relatively conserved *β*‐barrel core, whereas the major variations were concentrated in the surface‐exposed BC, DE, and GH loops. Experimental infection showed that BEV NM2407 was able to infect calves and induce mild clinical signs. Viral RNA was detected in nasal and rectal swabs as well as multiple tissues, with relatively higher levels in the trachea, lung, cecum, and rectum. Gross pathological changes were mainly characterized by intestinal hyperemia/congestion, most prominently in the jejunum. Histopathological examination revealed epithelial necrosis in the jejunum and rectum, together with lymphocyte depletion and necrosis in ileal lymphoid follicles. Overall, BEV NM2407 demonstrated infectivity in calves under experimental conditions. The observed viral shedding, tissue distribution, and localized pathological lesions provide further evidence of the pathogenic potential of BEV, expand current understanding of its molecular evolution and biological characteristics, and provide a basis for further investigations into the pathogenesis of BEV infection in cattle.

## 1. Introduction

Bovine diarrhea is endemic in dairy cattle herds across China and remains one of the major causes of growth retardation and mortality in calves, resulting in substantial economic losses to the livestock industry [1]. In recent years, bovine enterovirus (BEV) has been identified in cattle herds across multiple geographical regions of China in numerous epidemiological investigations, confirming its widespread circulation in Chinese cattle populations. Accordingly, the potential contribution of BEV to the pathogenesis of bovine diarrhea has gradually attracted increasing research attention [2, 3].

BEV is a member of the genus *Enterovirus* within the family *Picornaviridae* and is classified as a nonsegmented, single‐stranded positive‐sense RNA virus [4]. Previous studies have shown that BEV infection can induce a wide spectrum of clinical manifestations in cattle, including respiratory disease, enteric disease, and reproductive disorders [5]. In addition, some enteroviruses have been reported to exhibit neurotropic or neuroinvasive potential, suggesting that their tissue tropism may extend beyond the respiratory and gastrointestinal tracts; however, evidence for such properties in BEVs remains limited [6]. According to current nomenclature recommendations for enteroviruses and rhinoviruses, the genus *Enterovirus* includes *Enterovirus* A–H and J (EV‐A to EV‐J), as well as *Rhinovirus* A–C (RV‐A to RV‐C) [7]. Among these, *Enterovirus* E (EV‐E) comprises five subtypes (E1 to E5). The full‐length BEV genome is ~7.1–7.5 kb, with untranslated regions (UTRs) at the 5^′^ and 3^′^ termini, respectively, and a single open reading frame (ORF) in the central region that encodes a polyprotein. This polyprotein undergoes proteolytic cleavage to generate four structural proteins (VP1–VP4) and seven nonstructural proteins (2A–2C and 3A–3D) [8].

VP1 is a major capsid protein of BEV, and its amino acid variation may be associated with viral antigenicity and host interactions. Previous molecular classification studies have shown that capsid protein‐coding regions, including VP1, are useful for distinguishing BEV geno‐/serotypes. Therefore, the VP1 coding region is commonly used as an important molecular marker for BEV genotyping and phylogenetic analysis [9]. Multiple antigenic loop structures are distributed across the VP1 protein, among which the BC‐loop, DE‐loop, and GH‐loop are surface‐exposed antigenic loops recognized as key regions for neutralizing antibody recognition and antigenic variation [10]. Accumulating evidence has shown that these antigenic loops may display differential conservation and variation patterns in enteroviruses derived from different host species, suggesting their critical roles in cross‐species transmission and adaptation to novel hosts [11]. In addition, VP2 is another key viral capsid protein, and sequence analysis of the VP2 gene enables subtype identification of enteroviruses without the need for virus isolation [12]. Accordingly, VP1 and VP2 are frequently used in combination for the phylogenetic analysis and genotyping of enteroviruses.

Since the first isolation and identification of BEV, the virus has been successively reported in multiple countries, including Turkey, China, and Brazil [13–15]. With the growing number of global BEV isolates, the genomic diversity of BEV has continuously expanded, and its subtype composition has become increasingly complex, indicating that the virus has undergone persistent genetic variation and recombination events. Despite its broad geographic distribution and frequent detection in cattle, research on the pathogenicity of BEV remains relatively limited, and no universally accepted animal model is currently available. Although calves have traditionally been used for BEV pathogenesis studies, practical constraints have encouraged researchers to explore other BEV‐related experimental infection models, including neonatal mice and sheep [16, 17]. While these models have provided information on BEV replication, shedding, host responses, or pathological changes, they cannot fully recapitulate the natural infection process and tissue tropism of BEV in cattle because of marked differences in physiology and immunological background between model animals and the natural host. Furthermore, in recent years, BEV‐related enteroviruses and recombinant strains have been detected in nonbovine hosts, such as alpacas and sheep [18], suggesting possible cross‐host circulation and evolutionary complexity [19]. These findings not only deepen the understanding of the genetic and evolutionary complexity of BEV but also indicate that the virus may acquire novel host adaptability through mechanisms such as genetic recombination. However, there is still a lack of systematic studies evaluating the infectivity, tissue distribution, and clinicopathological changes associated with BEV infection in cattle. In addition, its potential cross‐host transmission risk and underlying mechanisms remain to be further explored. Against this background, in the present study, a BEV‐E4 strain was isolated from a fecal sample of a diarrheic calf in Inner Mongolia, China. Whole‐genome sequencing, phylogenetic analysis, and homologous recombination analysis were performed to characterize its genomic organization and evolutionary relationships. VP2 was included as an additional phylogenetic marker to support subtype assignment, whereas VP1 was selected for detailed structural analysis because it is the major surface‐exposed capsid protein associated with antigenicity and host interaction. Furthermore, an experimental infection model was established in Holstein calves, the natural host of BEV. Viral shedding was monitored continuously, viral RNA loads in various tissues were quantified, histopathological examination was performed to evaluate tissue changes and the pathogenic potential of the isolate under experimental conditions.

## 2. Materials and Methods

### 2.1. Clinical Specimens, Sample Processing

In July 2024, a total of seven fresh fecal samples were collected from 8‐month‐old diarrheic Holstein calves at a large‐scale dairy farm in Inner Mongolia, China. Briefly, each fecal sample was separately resuspended in 10 mM phosphate‐buffered saline (PBS, pH 7.4, Biosharp, Cat. No. BL302A) and thoroughly homogenized, then centrifuged at 4°C and 13,000 ×*g* for 10 min. The resulting supernatants were collected and equally divided into two aliquots: one aliquot was used for total nucleic acid extraction, and the other was used for virus isolation and identification. All samples were stored at −80°C prior to subsequent experiments.

### 2.2. Nucleic Acid Extraction, RT‐PCR, and qRT‐PCR

Total nucleic acids, including viral RNA and bacterial DNA, were extracted from pretreated fecal supernatants using the TIANamp Virus DNA/RNA Kit (TIANGEN, Cat. No. DP315) according to the manufacturer’s instructions. RT‐PCR amplification was performed using the HiScript II One Step RT‐PCR Kit (Dye Plus, Cat. No. P612‐01), and quantitative real‐time PCR (qRT‐PCR) was carried out using the HiScript II One Step qRT‐PCR Probe Kit (Cat. No. Q222‐01). Primers and probes for the detection of BEV [20], bovine viral diarrhea virus (BVDV) [21], bovine coronavirus (BCoV), and bovine rotavirus (BRV) [22], as well as primers for PCR detection of the *Escherichia coli* heat‐stable enterotoxin STa virulence gene [23], were adopted from previously published assays and synthesized by Sangon Biotech Co., Ltd. (Shanghai, China).

Primers and probes used in this study are listed in Table S1. First, qRT‐PCR was used to screen the seven fecal samples from diarrheic calves for BEV, and BEV‐positive fecal samples were selected for subsequent virus isolation based on the detection results. Representative qRT‐PCR validation results for the BEV assay controls and the index clinical sample are provided in Table S2. For each RT‐PCR, PCR, or qRT‐PCR assay, a previously confirmed positive sample or standard template was included as a positive control, and nuclease‐free water was used as a no‐template control. A negative extraction control was also included during assay validation.

### 2.3. Cell Culture and Virus Isolation

Madin‐Darby bovine kidney (MDBK, ATCC CCL‐22) cells were maintained in Dulbecco’s modified Eagle medium (DMEM, Gibco, Cat. No. 11995065) supplemented with 10% fetal bovine serum (FBS, Upsilon, Cat. No. FSA01‐500) at 37°C in a humidified incubator. The clarified fecal supernatants obtained were filtered through a 0.22 μm membrane filter and inoculated onto confluent MDBK cell monolayers. Cells were observed daily under an inverted microscope for the appearance of cytopathic effects (CPE). If no obvious CPE was observed, continuous blind passages were performed. When typical CPE reached ~80%, the infected cells and supernatants were subjected to three freeze–thaw cycles at −80°C, and the virus‐containing supernatants were collected. The harvested virus was further tested for major diarrhea‐associated pathogens (BEV, BVDV, BCoV, BRV, and *E. coli*) and stored at −80°C as virus stocks.

### 2.4. Plaque Purification and Preparation of Samples for Transmission Electron Microscopy

The virus‐containing culture supernatant was subjected to three freeze–thaw cycles and centrifuged at 12,000 ×*g* for 10 min at 4°C. The clarified supernatant was collected for plaque purification. MDBK cells were seeded in six‐well plates and grown to ~90%–100% confluence. Serial 10‐fold dilutions of the virus suspension were prepared and inoculated onto the cell monolayers, followed by incubation at 37°C for 1 h to allow viral adsorption. After adsorption, the inoculum was removed and cells were overlaid with a mixture of 3% low‐melting‐point agarose (Thermo Fisher Scientific, Cat. No. R0801) and DMEM. The plates were incubated at 37°C in a humidified atmosphere and monitored daily for plaque formation. When well‐isolated plaques with clear boundaries and typical morphology appeared, individual plaques were carefully picked and transferred into microcentrifuge tubes containing a small volume of DMEM. The recovered virus was used to re‐infect MDBK cells for subsequent serial passages to obtain a purified viral clone. For plaque visualization, the semi‐solid overlay was gently removed, and the cells were fixed with 4% paraformaldehyde (Solarbio, Cat. No. P1110) at room temperature for 20 min. After air‐drying, the cells were stained with 1 mL staining solution containing 0.1% crystal violet (Biosharp, Cat. No. BL802A) for 15 min, followed by rinsing and drying.

For electron microscopy, 1 mL of virus suspension was inoculated into T75 flasks containing MDBK cells. When CPE reached ~80%, the culture supernatant was harvested. The suspension was centrifuged at 12,000 ×*g* for 10 min at 4°C to remove cellular debris. The clarified supernatant was subsequently ultracentrifuged at 151,000 ×*g* for 5 h at 4°C. The viral pellet was resuspended in 0.2 mL PBS. A 10 μL aliquot was applied onto a 300‐mesh carbon‐coated copper grid and allowed to adsorb for 10 min. After thorough washing with distilled water, the grid was negatively stained twice with 2% phosphotungstic acid (Biosharp, Cat. No. BL2316A) for 10 s each time. The grids were air‐dried and examined using a Hitachi HT7800 transmission electron microscope (Hitachi, Japan) at an accelerating voltage of 80 kV.

### 2.5. Indirect IFA

MDBK cells were infected with the virus, and uninfected cells were used as negative controls. At 24 h postinfection (hpi), the inoculum was removed and the cells were fixed with 4% paraformaldehyde at room temperature for 20 min. After blocking with 5% bovine serum albumin (BSA, Biosharp, Cat. No. A8010) for 30 min, the cells were incubated overnight at 4°C with rabbit anti‐BEV VP2 polyclonal antibody (PcAb; prepared in our laboratory; detailed methods are provided in the Supporting Information). After washing, FITC‐conjugated goat anti‐rabbit IgG (H&L) secondary antibody (Abcam, Cat. No. AB6717) was added and incubated at 37°C for 1 h in the dark. Cell nuclei were counterstained with 4^′^,6‐diamidino‐2‐phenylindole (DAPI; Merck, Cat. No. D9542) for 10 min. After each step, the cells were washed three times with phosphate‐buffered saline containing 0.05% Tween‐20 (PBST) for 5 min each. Fluorescence signals were observed and captured using a fluorescence inverted microscope (ZEISS, Germany).

### 2.6. TCID_50_ Determination

The 50% tissue culture infectious dose (TCID_50_) of the virus was determined using 96‐well plates. The virus stocks were subjected to 10‐fold serial dilutions and inoculated onto MDBK cells. After incubation for 96 hpi, CPE was recorded, and viral titers were calculated using the Reed‐Muench method and expressed as TCID_50_/mL [24].

### 2.7. Whole‐Genome Sequencing

MDBK cells and culture supernatants showing more than 80% CPE were subjected to three freeze–thaw cycles at −80°C. The samples were then centrifuged at 12,000 ×*g* for 10 min at 4°C, and the supernatants were collected for RNA extraction. Viral RNA was extracted using the TIANamp Virus DNA/RNA Kit, and the extracted RNA was used for RNA library construction. According to the protocol provided by Magigene (China), next‐generation sequencing (NGS) was performed on the Illumina NovaSeq 6000 platform to obtain the complete genome sequence of the isolated strain. Raw reads were quality‐filtered and assembled into contigs, and the resulting viral genome sequence was confirmed by comparison with sequences in the NCBI nucleotide database. The consensus genome was then generated and annotated for UTRs, ORF boundaries, and coding regions.

### 2.8. Phylogenetic Analysis

To characterize the genetic and evolutionary features of the isolate identified in this study, enteroviruses from different species were retrieved from the NCBI nucleotide database, and a total of 37 representative enterovirus full‐length genome sequences, together with the corresponding VP1 and VP2 coding sequences, were included in the final dataset. Multiple sequence alignments were conducted using MEGA 11.0 software. For each sequence dataset, the optimal nucleotide substitution model was selected via the built‐in Model Selection module in MEGA. Phylogenetic trees were subsequently generated using the Maximum Likelihood (ML) method based on the corresponding optimal model for each alignment. The topological reliability of the phylogenetic trees was assessed by bootstrap analysis with 1000 replicates, and bootstrap support values were labeled at each corresponding node. The final trees were visualized and preliminarily annotated using Interactive Tree Of Life (iTOL, version 7.4.2, https://itol.embl.de/). Sequence labels in the phylogenetic trees were presented as accession number, subtype, and country of origin. The list of reference sequences used for phylogenetic and recombination analyses, including accession number, subtype/species name, host, and country, is provided in Table S3.

### 2.9. Homologous Recombination Analysis

The sequence dataset used for homologous recombination analysis included the BEV NM2407 sequence obtained in this study, while the other sequences were the same as those used for phylogenetic analysis, resulting in a total of 37 full‐genome sequences. The multiple sequence‐aligned complete genomes were imported into the Recombination Detection Program (RDP, version 4.101), and homologous recombination analysis was conducted under default settings using seven algorithms implemented in the software (RDP, GENECONV, BootScan, MaxChi, Chimaera, SiScan, and 3Seq) for cross‐validation. A recombination event was considered credible only when it was supported by at least five of the seven methods, with a Bonferroni‐corrected *p*‐value <1.0 × 10^−6^. The recombination event confirmation table generated by RDP was exported, and the recombination score reported by the software was recorded to quantify the strength of the recombination signal. Pairwise similarity plots were also generated to visualize changes in sequence similarity between the query sequence (the isolate obtained in this study) and the putative major/minor parental strains across different genomic regions. The “major” and “minor” parental sequences inferred by RDP were interpreted as the closest available related sequences within the analyzed dataset, rather than definitive biological parental strains. Putative recombination signals identified by RDP were further validated using SimPlot version 3.5.1 to support the recombination inference and approximate breakpoint positions, rather than to determine definitive biological parental strains. SimPlot analyses were performed with a sliding window size of 200 bp and a step size of 20 bp, with all other parameters set to software defaults. Final breakpoint positions were estimated based on segmental shifts in similarity patterns observed in the SimPlot similarity curves. The full list of reference sequences included in the recombination dataset is provided in Table S3.

### 2.10. VP1 Amino Acid Sequence Alignment and Three‐Dimensional Structural Analysis

To characterize amino acid variations in the VP1 protein of the isolate obtained in this study (GenBank accession number: PV032957.1) and to explore their potential structural and functional significance, a comparative dataset of enterovirus VP1 amino acid sequences from different species was constructed. In addition to BEV NM2407, the dataset included one bovine EV‐E4 strain, two bovine EV‐F1 strains, two human EV‐C strains, and two human EV‐D representative strains. Multiple sequence alignment of VP1 amino acid sequences was performed in MEGA version 11.0 using ClustalW with default parameters. The alignment output was imported into ESPript version 3.2 for visualization and conservation analysis. Particular attention was paid to the surface‐exposed antigenic loop regions of VP1, and amino acid differences and conservation patterns within the BC‐loop, DE‐loop, and GH‐loop were compared among strains from different host origins.

To map sequence variation onto a spatial structural framework, the three‐dimensional (3D) structure of VP1 from the present isolate was predicted using AlphaFold 3. A previously published high‐resolution crystal structure of enterovirus A71 (EV‐A71) VP1 (PDB ID: 3VBF) was used as a reference for loop boundary definition. Structural visualization, superposition, and loop annotation were conducted in PyMOL (version 3.0.3). The predicted VP1 structure was aligned to the EV‐A71 VP1 reference structure, and the corresponding BC‐loop, DE‐loop, and GH‐loop boundaries were defined based on the exact loop positions in the reference crystal structure. Key amino acid differences identified from the multiple sequence alignment were then mapped onto the solvent‐accessible surface of the predicted VP1 structure, to compare the spatial localization and conformational variation of these critical antigenic loop regions among enteroviruses from different host origins.

### 2.11. Animal Experiment

All animal experimental procedures were reviewed and approved by the Animal Welfare and Ethics Committee of Inner Mongolia Agricultural University (Approval No. NND2022114), and were conducted in strict accordance with applicable national and institutional animal welfare regulations, as well as the ARRIVE 2.0 reporting guidelines.

A total of six clinically healthy, colostrum‐fed 4‐week‐old Holstein calves were obtained from a large‐scale dairy farm with no reported history of BEV‐associated disease. Prior to enrollment, each calf underwent eligibility screening. Nasal and rectal swabs were collected and tested for BEV RNA by strain‐specific qRT‐PCR, and serum samples were collected and examined for BEV antibodies using an indirect ELISA to exclude possible prior exposure or interference from maternal antibodies. Only calves that were negative for BEV RNA and BEV antibodies, and without clinical signs of diarrhea or other systemic illness, were included in the subsequent experimental infection.

#### 2.11.1. Animal Experiment Design

**Figure 1:**
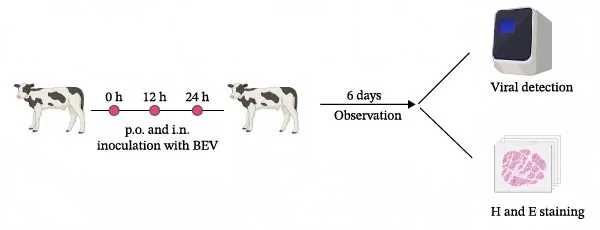
Schematic overview of the experimental design for the BEV infection model in calves. Healthy Holstein calves were inoculated with BEV via combined intranasal inoculation and oral gavage at 0, 12, and 24 h (three administrations in total). Calves were monitored for clinical signs for 6 days following inoculation. At 6 dpi, calves were euthanized for necropsy, and tissues were collected for viral load determination and histopathological examination using hematoxylin and eosin (H&E) staining to evaluate tissue changes. The 6‐dpi necropsy time point was selected based on the results of a preliminary pilot experiment.

As shown in Figure 1, six calves were randomly allocated to the BEV challenge group and the mock‐inoculated negative control group (*n* = 3 calves per group). Calves in the challenge group were fasted for 12 h, with free access to water, before the first inoculation and were challenged with BEV NM2407 on three occasions, at 0, 12, and 24 h, via combined intranasal inoculation (10 mL) and oral gavage (20 mL) routes at a titer of 10^9.3^ TCID_50_/mL. Calves in the control group received the same volume of serum‐free DMEM via the same routes and schedule. The repeated inoculation design was used to maximize exposure and ensure establishment of infection under experimental conditions, rather than to fully mimic a natural single‐exposure event.

Before the formal animal experiment, a preliminary pilot experiment was conducted to help determine the inoculation design and necropsy time point. Based on viral shedding, clinical observations, and tissue viral RNA detection in the pilot experiment, 6 days postinoculation (dpi) was selected as the necropsy time point for the formal experiment. This timing was also informed by acute‐phase tissue sampling and necropsy time points used in calf infection models of BRV, BCoV, and BVDV [25–26].

Following inoculation, all calves were monitored twice daily at fixed time points for clinical signs, including general condition, milk intake, respiratory status, and fecal consistency. Nasal and rectal swabs were collected daily for viral RNA load quantification by qRT‐PCR. Calf appetite was assessed based on the amount of milk intake.

At 6 dpi, all calves were placed under deep anesthesia and then euthanized by intravenous administration of a saturated potassium chloride solution, followed by systematic necropsy in accordance with the approved animal ethics protocol. Target tissues were collected, with one portion stored at −80°C for viral RNA load detection and virological analysis, and another duplicate sample fixed in 4% paraformaldehyde for histopathological examination.

#### 2.11.2. Quantification of Viral RNA Loads in Nasal and Rectal Swabs

Viral RNA loads of BEV NM2407 in nasal and rectal swabs were quantified separately by a BEV qRT‐PCR assay targeting the conserved 5^′^UTR at 1 day before inoculation (0 dpi) and daily from 1 to 6 dpi. These data were used to characterize the respiratory and gastrointestinal shedding dynamics of BEV NM2407 in experimentally challenged calves. Nasal and rectal swabs were processed and tested individually prior to RNA extraction. Each swab was suspended in PBS, clarified by centrifugation, and the resulting supernatant was used for RNA extraction. BEV qRT‐PCR quantification was performed according to a previously published TaqMan probe‐based assay [20]. In the present study, copy‐number estimation followed the published copy‐number calculation strategy and assay interpretation criteria. Samples with Cq values ≤36 were considered positive, whereas samples with Cq values >36 were considered negative. According to the published assay, the analytical detection limit was 10 copies/μL [20].

#### 2.11.3. Gross Pathology and Viral Tissue Distribution of BEV

At 6 dpi, all calves in the BEV NM2407 challenge group and the mock‐inoculated negative control group were deeply anesthetized and euthanized by intravenous administration of a saturated potassium chloride solution in accordance with the approved animal ethics protocol. Systematic necropsy was then performed immediately. Heart, liver, spleen, lung, kidney, trachea, duodenum, jejunum, ileum, cecum, colon, and rectum were collected in duplicate. One sample of each tissue was snap‐frozen in liquid nitrogen and stored at −80°C for viral load quantification, whereas the duplicate sample was fixed histopathological examination. Viral loads of BEV NM2407 in tissue samples were quantified using the same BEV qRT‐PCR assay targeting the conserved 5^′^UTR as that used for swab samples [20]. Copy number estimation for tissue samples followed the same previously published assay and interpretation criteria used for swab samples. Samples with Cq values ≤36 were considered positive, whereas samples with Cq values >36 were considered negative. According to the published assay, the analytical detection limit was 10 copies/μL [20]. These data were used to characterize the tissue distribution of BEV NM2407 in experimentally challenged calves.

#### 2.11.4. Histopathological Examination

Tissue samples, including respiratory tissues (trachea and lung), gastrointestinal tissues (duodenum, jejunum, ileum, cecum, colon, and rectum), and major visceral organs (heart, liver, spleen, and kidney), were fixed in 4% paraformaldehyde for 24 h at 37°C immediately after collection. Fixed tissues were processed through a standard histopathological workflow: graded ethanol dehydration, xylene clearing, and paraffin wax embedding. Paraffin blocks were cut into 4 μm‐thick consecutive sections, which were then stained with hematoxylin and eosin (H&E) following a standardized protocol. Histopathological lesions in each section were observed, evaluated, and documented under a bright‐field light microscope.

### 2.12. Statistical Analysis

**Figure 2:**
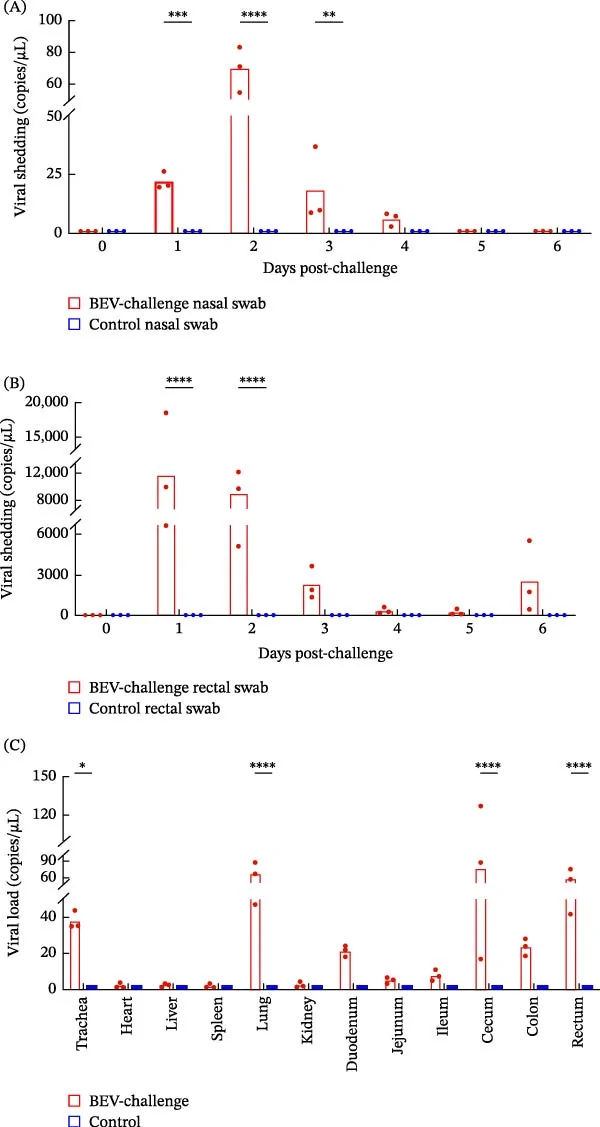
Viral RNA loads of BEV NM2407 in experimentally challenged calves. (A) Viral RNA loads in nasal swabs collected from challenged and mock‐inoculated calves from 0 to 6 dpi. (B) Viral RNA loads in rectal swabs collected from challenged and mock‐inoculated calves from 0 to 6 dpi. (C) Viral RNA loads in tissues collected at 6 dpi. Viral RNA loads are expressed as copies/μL. Individual data points are shown, and bars indicate mean ± SD. Statistical significance was analyzed by two‐way ANOVA followed by Šídák’s multiple comparisons test to compare challenged and mock‐inoculated calves at the corresponding time point or tissue.  ^∗^ *p* < 0.05,  ^∗∗^ *p* < 0.01,  ^∗∗∗^ *p* < 0.001,  ^∗∗∗∗^ *p* < 0.0001.

All statistical analyses were performed using GraphPad Prism 10.4.1 software. Data are presented as mean ± SD. Data shown in Figure 2A–C were analyzed by two‐way analysis of variance (two‐way ANOVA), followed by Šídák’s multiple comparisons test. For nasal and rectal swab viral RNA load analyses, comparisons were made between the BEV‐challenge and control groups at each time point. For tissue viral RNA load analysis, comparisons were made between the BEV‐challenge and control groups within each tissue. These comparisons were used to determine whether viral RNA loads in BEV‐challenged calves were above the background levels observed in mock‐inoculated controls, rather than to compare viral loads among different time points or among different tissues. Individual calves were analyzed as separate biological replicates, and swab samples were not pooled.

## 3. Results

### 3.1. Isolation and Identification of the BEV NM2407 Strain

qRT‐PCR targeting the conserved 5^′^UTR detected BEV RNA in all seven clinical diarrheic fecal samples, and the corresponding Cq values are shown in Table S2. Based on the qRT‐PCR screening results, three BEV‐positive fecal samples with relatively low Cq values were selected for virus isolation in MDBK cells. All three samples induced similar CPE after inoculation onto MDBK cells, characterized by cell rounding/shrinkage, increased refractivity, aggregation, focal monolayer disruption, and partial detachment. Subsequently, based on the CPE appearance and BEV nucleic acid detection results, one BEV‐positive cell culture showing evident CPE was selected as a representative virus isolate for subsequent virus purification, identification, whole‐genome sequencing, and biological characterization.

**Figure 3:**
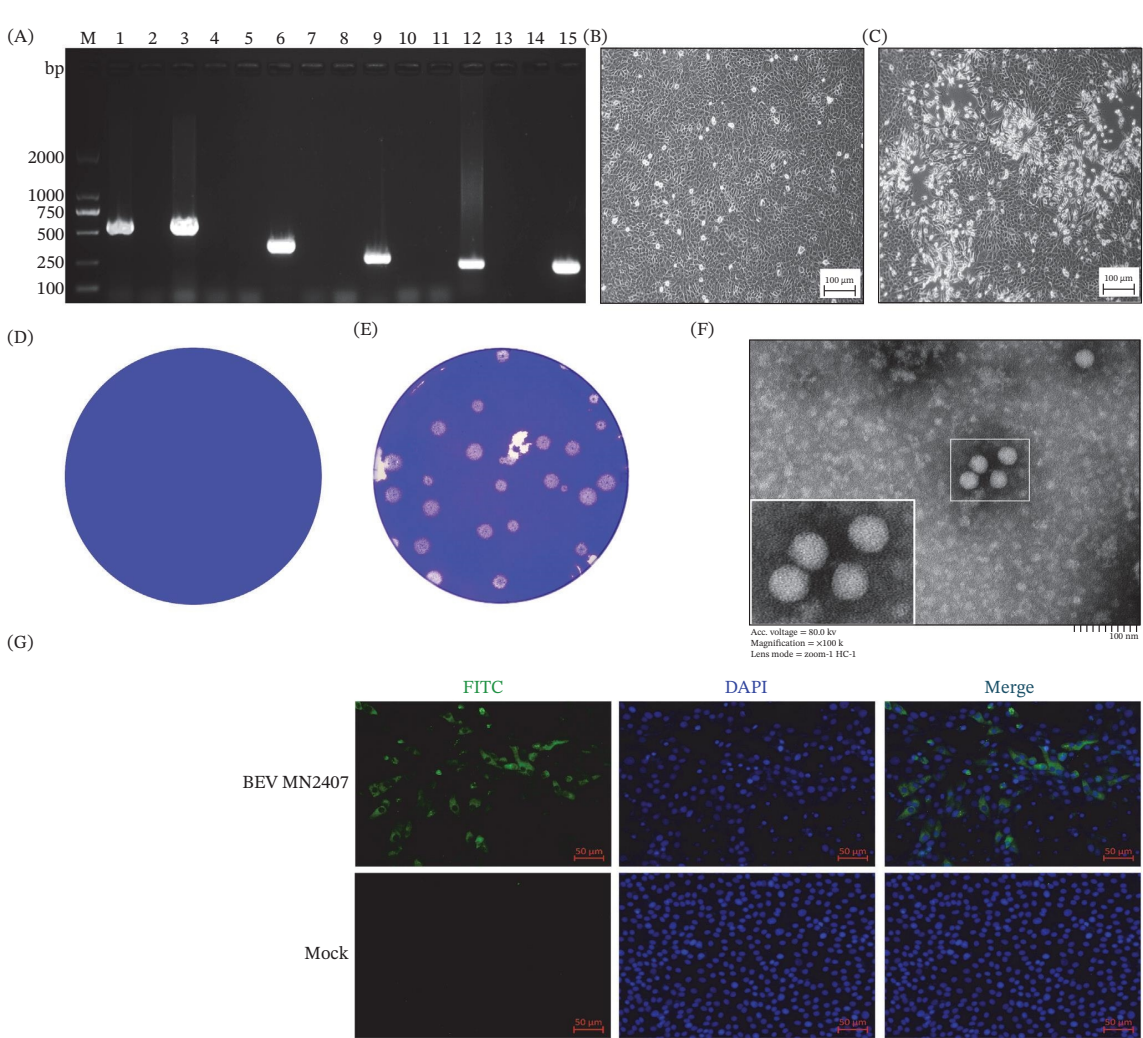
Isolation and identification of BEV NM2407. (A) RT‐PCR/PCR screening of the clinical diarrheic fecal sample for BEV, BRV, BVDV, BCoV, and the *E. coli* STa virulence gene, together with the corresponding assay controls. Lane M: DL 2000 bp DNA marker; lanes 1–3: BEV assay (1, clinical sample; 2, no‐template control; 3, BEV positive control); lanes 4–6: BRV assay (4, clinical sample; 5, no‐template control; 6, BRV positive control); lanes 7–9: BVDV assay (7, clinical sample; 8, no‐template control; 9, BVDV positive control); lanes 10–12: BCoV assay (10, clinical sample; 11, no‐template control; 12, BCoV positive control); lanes 13–15: *E. coli* STa virulence gene assay (13, clinical sample; 14, no‐template control; 15, positive control). (B) Mock‐inoculated MDBK cells. (C) CPE in MDBK cells infected with BEV NM2407 at 48 hpi. (D) Plaque assay negative control (mock). (E) Plaques formed by BEV NM2407 after plaque purification. (F) Negative‐stain transmission electron microscopy image showing nonenveloped, rounded BEV NM2407 particles. (G) IFA of MDBK cells infected with BEV NM2407 using an in‐house anti‐BEV‐VP2 PcAb (FITC, green); nuclei were counterstained with DAPI (blue). Scale bars: 100 μm (B and C), 100 nm (F), and 50 μm (G).

In this sample, RT‐PCR assays for other common bovine enteric pathogens, including BVDV, BCoV, BRV, and the *E. coli* STa virulence gene, were negative, indicating no detectable co‐infection with these tested pathogens. The corresponding positive and no‐template controls performed as expected in each assay (Figure 3A). No CPE was observed in mock‐inoculated MDBK cells (Figure 3B), whereas MDBK cells inoculated with the treated BEV‐positive sample developed clear CPE at 48 hpi, characterized mainly by cell rounding/shrinkage, increased refractivity, aggregation, focal monolayer disruption, and partial detachment (Figure 3C). During serial passaging, the onset of CPE became earlier. By passage 10, CPE began to appear at ~12 hpi and then gradually became more severe, suggesting that the virus replicated stably in MDBK cells. After three rounds of plaque purification, no plaques were observed in the mock control (Figure 3D), whereas uniform round plaques with clear boundaries were obtained from the BEV isolate (Figure 3E). Negative‐stain transmission electron microscopy of the purified viral preparation revealed nonenveloped, rounded viral particles ~20–30 nm in diameter (Figure 3F). IFA using an in‐house rabbit polyclonal antibody against BEV‐VP2 showed strong cytoplasmic fluorescence in infected MDBK cells, while no specific signal was detected in uninfected controls (Figure 3G), confirming the identity of the isolate as BEV. The specificity of the in‐house rabbit anti‐BEV VP2 polyclonal antibody was validated by Western blot and ELISA (Figure S1). The antibody exhibited strong specificity and high titer, confirming its suitability for immunofluorescence assays (IFAs). The viral titer in MDBK cells was determined by TCID_50_ assay (eight replicates per 10‐fold dilution) and calculated using the Reed‐Muench method, reaching 10^9.3^ TCID_50_/mL. Collectively, BEV RNA was first detected by qRT‐PCR in seven fecal samples from diarrheic calves, and three samples with relatively low Cq values were selected for virus isolation. Because the three samples induced largely similar CPE in MDBK cells, one BEV‐positive cell culture showing evident CPE was ultimately selected for subsequent whole‐genome sequencing and systematic analysis. This representative isolate was designated BEV NM2407.

### 3.2. Complete Genome Characterization and Phylogenetic Analysis

The complete genome of BEV NM2407 was 7414 nt in length, comprising an 814‐nt 5^′^UTR, a 6525‐nt ORF, and a 75‐nt 3^′^UTR. The ORF encodes a 2175‐amino‐acid polyprotein with the typical picornavirus organization, including the P1 capsid region (VP4–VP2–VP3–VP1) and the P2/P3 nonstructural regions (2A–2B–2C and 3A–3B–3C–3D), consistent with previously described Enterovirus E genomes.

**Figure 4:**
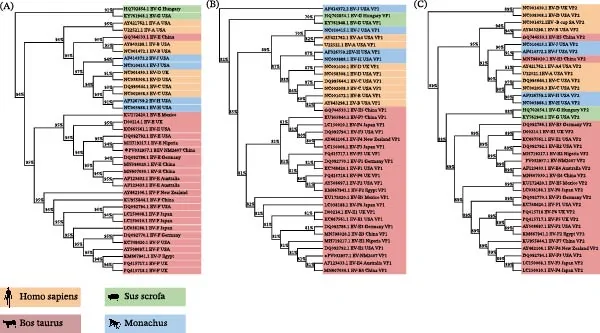
Phylogenetic analysis of BEV NM2407 based on the complete genome and VP1/VP2 coding sequences. (A) Complete‐genome phylogeny. (B) VP1–based phylogeny. (C) VP2–based phylogeny. Sequence labels are shown in simplified format (accession number, subtype/species designation, and country). Full sequence information is provided in Table S3.

Across all three phylogenetic trees, BEV NM2407 (PV032957.1) consistently clustered within the EV‐E clade and grouped with EV‐E4 reference strains (Figure 4A–C), supporting its subtype assignment as EV‐E4. The three phylogenetic trees showed concordant topologies and effectively separated EV‐E from the other enterovirus species included in this study at the species and subtype levels, providing a reliable framework for subsequent evolutionary analysis. VP2–based phylogeny showed a clustering pattern highly concordant with the complete genome and VP1 trees, further supporting the subtype identification of BEV NM2407. Considering that VP1 is the major surface‐exposed capsid protein closely associated with viral antigenicity and host interactions, subsequent comparative structural analyses were mainly focused on this protein.

### 3.3. Homologous Recombination Analysis

**Figure 5:**
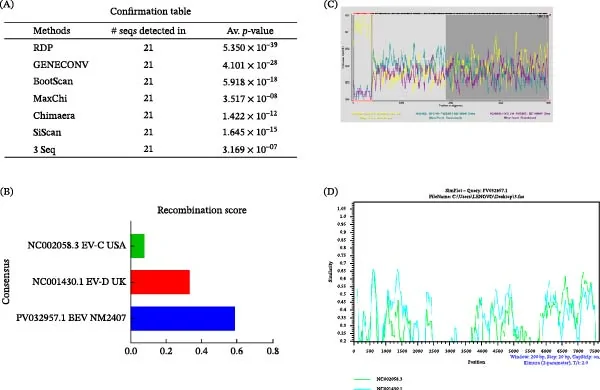
Homologous recombination analysis of BEV NM2407. (A) Statistical support for the putative recombination event identified by RDP. (B) Recombination score. (C) Pairwise similarity plot of BEV NM2407 against the putative parental lineages. (D) Schematic overview of the inferred breakpoint positions across the genome.

A strong recombination signal was detected in BEV NM2407, with recombinant regions showing the highest sequence similarity to reference lineages of EV‐C and EV‐D. The putative event was supported by all seven methods implemented in RDP with *p* < 1.0 × 10^−6^ (Figure 5A) and yielded a recombination score of 0.589 (Figure 5B). Pairwise similarity scanning revealed a typical segment‐switching pattern across the BEV NM2407 genome when compared with the putative parental lineages (Figure 5C). Combined breakpoint inference identified two major breakpoints at ~1950 nt and 5850 nt (Figure 5D), partitioning the genome into three segments. The 1–1950 nt region showed the highest similarity to a putative minor parent from the EV‐C lineage (NC002058.3, USA), whereas the 1951–5850 nt region was most similar to a putative major parent from the EV‐D lineage (NC001430.1, UK). The 5851 nt to the 3^′^ end again exhibited higher similarity to EV‐C lineage sequences. Collectively, these results support the presence of recombination signals in BEV NM2407 and indicate that some genomic regions are more closely related to EV‐C‐ and EV‐D‐related reference lineages within the analyzed dataset, highlighting the genetic mosaicism and evolutionary complexity of enteroviruses. The EV‐C‐ and EV‐D‐related sequences identified in the RDP and SimPlot analyses were interpreted as the closest available matches within the analyzed dataset rather than definitive biological parental strains.

### 3.4. VP1 Amino Acid Alignment and Three‐Dimensional Structural Analysis

**Figure 6:**
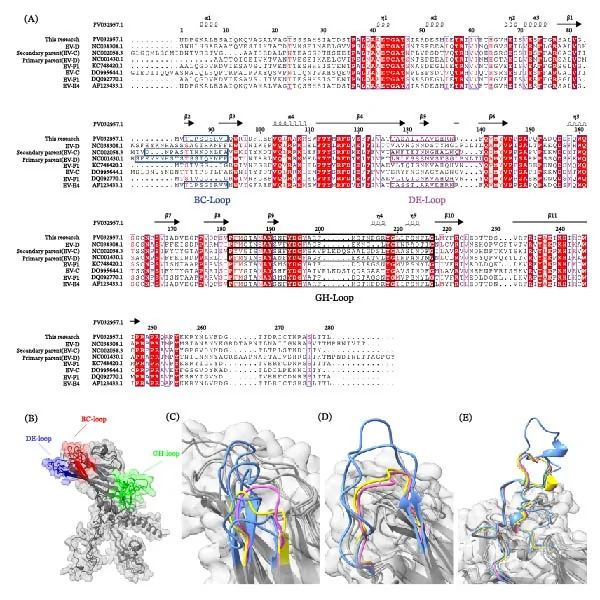
VP1 amino acid sequence alignment and structural comparison of surface‐exposed loop regions. (A) Multiple sequence alignment of VP1 proteins from BEV NM2407 and representative enteroviruses. Conserved residues and secondary‐structure elements are indicated, and the BC‐loop, DE‐loop, and GH‐loop are marked. (B) Three‐dimensional model of VP1 showing the surface localization of the BC‐loop (red), DE‐loop (blue), and GH‐loop (green). (C–E) Structural superposition of VP1 showing the BC‐loop (C), DE‐loop (D), and GH‐loop (E) in BEV NM2407 and representative reference strains. In Panels C–E, colored ribbons indicate BEV NM2407 (blue), the bovine EV‐E4 reference strain (yellow), the human EV‐C reference strain (magenta), and the human EV‐D reference strain (gray).

Multiple sequence alignment showed that the VP1 *α*‐helical and *β*‐strand core scaffold of BEV NM2407 is highly conserved relative to the reference enteroviruses, with only sporadic, scattered amino acid substitutions across the structural core. In contrast, sequence divergence was predominantly concentrated in three surface‐exposed loop regions, namely the BC‐loop, DE‐loop, and GH‐loop (Figure 6A). Specifically, the loop boundaries of BEV NM2407 were mapped to BC‐loop (85–93 aa), DE‐loop (127–138 aa), and GH‐loop (182–217 aa), and these three regions showed identical loop lengths and boundary positions compared with the bovine EV‐E4 reference strain. By comparison, the corresponding loop regions in the human EV‐C reference strain were mapped to BC‐loop (91–106 aa), DE‐loop (aa 140–153), and GH‐loop (197–238 aa), whereas those in the human EV‐D reference strain were BC‐loop (78–96 aa), DE‐loop (130–146 aa), and GH‐loop (190–225 aa). Notably, both human enterovirus references exhibited distinct loop lengths and boundary positions relative to BEV NM2407.

Three‐dimensional structural mapping and superposition further demonstrated that the BC‐loop, DE‐loop, and GH‐loop are fully exposed on the VP1 surface, corresponding to the hypervariable regions identified in the sequence alignment (Figure 6B). Structural comparison indicated that the BC‐loop conformation of BEV NM2407 more closely resembled that of the bovine EV‐E4 reference strain, whereas clear differences were observed relative to the human EV‐C and EV‐D strains in terms of loop extension and spatial orientation of side chains (Figure 6C). Among the three loops, the DE‐loop showed the most pronounced conformational divergence: the spatial bending trajectory of the BEV NM2407 DE‐loop differed from the more linearly extended conformation observed in the human enterovirus references and also differed from the folding mode of the bovine EV‐E4 reference (Figure 6D). In contrast, the overall backbone architecture of the GH‐loop was largely conserved across BEV NM2407 and the reference strains, with only minor positional differences confined to the distal flexible terminus of the loop (Figure 6E).

### 3.5. Pathogenicity of BEV NM2407 Strain

#### 3.5.1. Clinical Signs

All calves were tested for BEV‐specific antibodies using indirect ELISA prior to experimental infection, and all animals were confirmed to be seronegative (Figure S2), ensuring the absence of pre‐existing BEV exposure.

**Figure 7:**
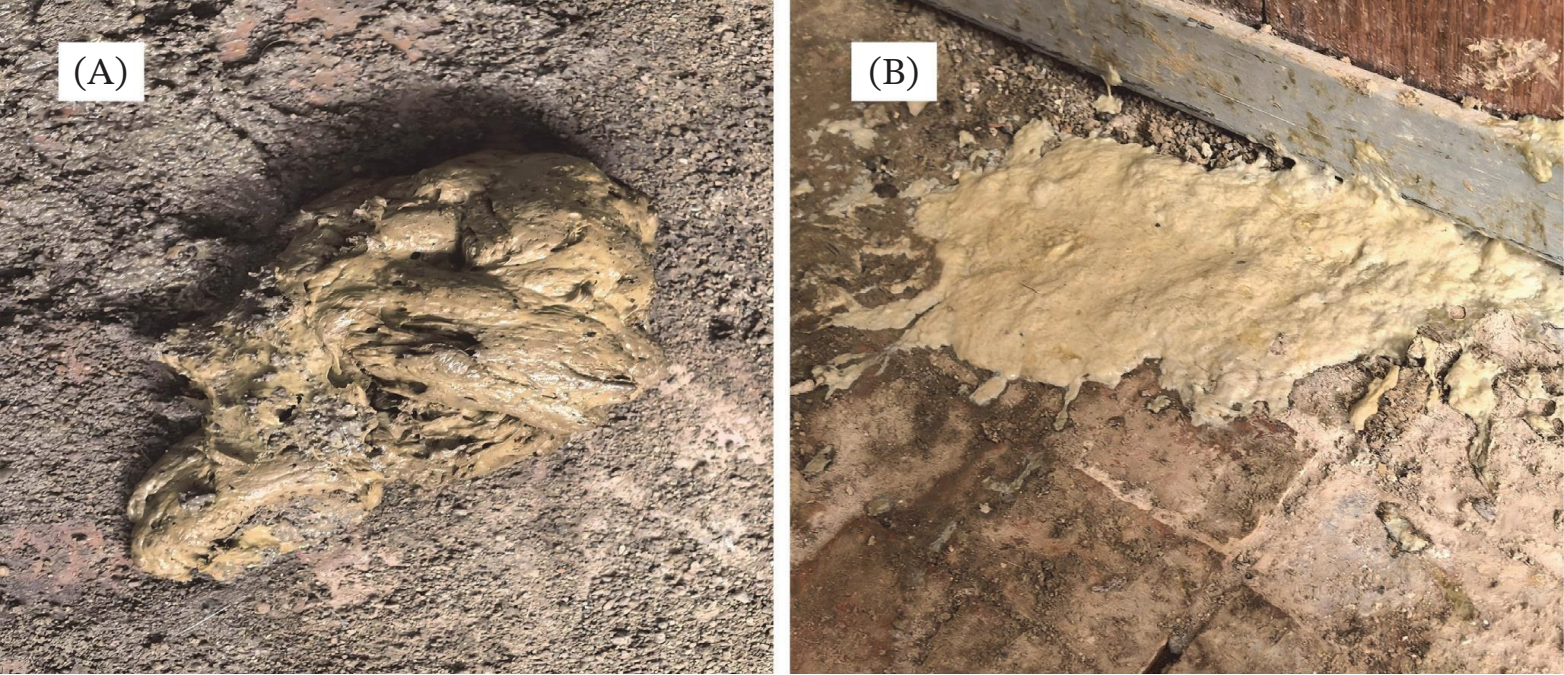
Representative fecal changes in calves following BEV NM2407 challenge. (A) Well‐formed feces from a mock‐inoculated control calf during the observation period. (B) Loose, pasty feces observed in a BEV NM2407‐challenged calf at 2 dpi.

Compared with mock‐inoculated controls, calves in the BEV NM2407‐challenged group showed generally mild clinical changes, including lethargy and reduced milk intake, whereas no abnormal signs were observed in the control group. Throughout the experiment, control calves maintained normal, well‐formed feces (Figure 7A). Importantly, clinical outcomes were variable among challenged calves: two of three challenged calves developed diarrhea starting at 2 dpi, with feces changing from well‐formed to loose, pasty stools; a small amount of mucus was occasionally observed (Figure 7B). Diarrhea persisted for 3–4 days and then gradually resolved. The remaining challenged calf exhibited mild lethargy and reduced milk intake without diarrhea.

#### 3.5.2. Gross Examination

**Figure 8:**
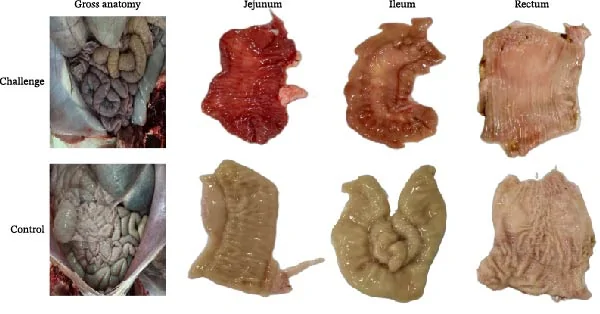
Representative gross findings in the abdominal cavity and intestinal tissues from BEV NM2407‐challenged and mock‐inoculated calves at 6 dpi. Representative gross images of the abdominal cavity, jejunum, ileum, and rectum collected at necropsy from challenged and control calves.

During gross examination, the jejunum, ileum, and rectum of BEV NM2407‐challenged calves exhibited mucosal hyperemia/congestion compared with mock‐inoculated controls. These findings were most evident in the jejunum, whereas no obvious gross abnormalities were observed in the other examined tissues (Figure 8).

#### 3.5.3. Viral Shedding Dynamics and Tissue Distribution

All calves tested negative prior to inoculation. In the challenge group, BEV RNA was detected in both nasal and rectal swabs after inoculation, and all challenged calves showed detectable shedding during the observation period, although individual variation was observed in the timing and magnitude of shedding. Nasal shedding increased after inoculation and peaked at 2 dpi, followed by a progressive decline to baseline levels by 5–6 dpi (Figure 2A). Rectal shedding appeared earlier and was generally higher than nasal shedding; rectal viral RNA loads peaked at 1 dpi, remained relatively high at 2–3 dpi, and then decreased from 4 to 5 dpi, with a secondary increase observed at 6 dpi (Figure 2B). In contrast, nasal and rectal swabs from mock‐inoculated controls remained at or near the assay detection limit throughout the observation period (Figure 2A,B). BEV RNA was detected in multiple tissues from the challenge group at 6 dpi (Figure 2C), with relatively higher viral RNA loads in the trachea, lung, cecum, and rectum than in other tissues. Viral RNA loads in the heart, liver, spleen, kidneys, small intestinal segments, and colon were comparatively lower, and no detectable signal above the assay threshold was observed in tissues from control calves (Figure 2C).

#### 3.5.4. Histopathological Changes

**Figure 9:**
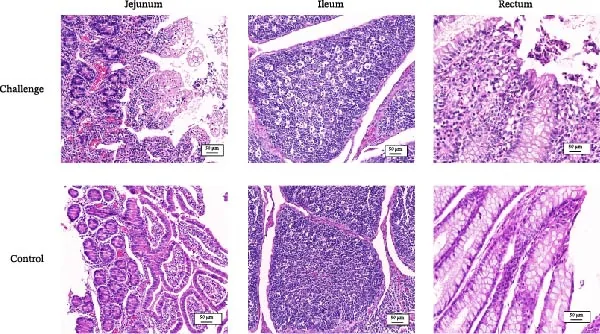
Representative H&E‐stained sections of intestinal tissues from BEV NM2407‐challenged and mock‐inoculated calves at 6 dpi. Representative sections of the jejunum, ileum, and rectum from challenged and control calves. Scale bars, 50 μm.

Histologic assessment showed that the jejunum of BEV NM2407‐challenged calves exhibited multifocal areas with necrosis of the superficial epithelium of the villi (Figure 9). In the ileum of challenged calves, there was a reduction and necrosis in the lymphocytes from the lymphoid follicles. The rectum of challenged calves showed areas of necrosis of the superficial epithelium of the rectal mucosa. No obvious histopathological lesions were observed in the other examined tissues.

## 4. Discussion

In the present study, BEV RNA was detected in all seven fecal samples collected from diarrheic Holstein calves on a cattle farm in Inner Mongolia, China. Based on the qRT‐PCR results, three BEV‐positive fecal samples with relatively low Cq values were selected for virus isolation. All three samples induced similar CPE after inoculation onto MDBK cells, and one BEV‐positive cell culture showing more evident CPE was ultimately selected for subsequent identification and whole‐genome sequencing. The obtained isolate was designated BEV NM2407. Notably, the representative sample tested negative for BVDV, BCoV, BRV, and the *E. coli* STa virulence gene, suggesting that no co‐infection with these common enteric pathogens was detected within the scope of the assays used in this study. However, because the original field case was not comprehensively screened for other bacterial or protozoal enteric pathogens, the possibility that other undetected factors contributed to diarrhea in the source animal cannot be completely excluded.

Regarding its in vitro replication characteristics, BEV NM2407 replicated stably in MDBK cells, with a viral titer reaching 10^9.3^ TCID_50_/mL, suggesting that this isolate had good adaptation to MDBK cells and a high infectious titer. Previous studies have shown that different BEV isolates may exhibit variable infectious titers in cell culture. The BEV‐F1 isolate HB19‐1 propagated well in MDBK cells and reached a peak titer of ~10^6^ TCID_50_/mL at 36 hpi [16], whereas the recombinant BEV HeN‐2022 reached a peak titer of ~10^5^ TCID_50_/mL in MDBK cells at 36 hpi [17]. Compared with these reports, the relatively high titer obtained for BEV NM2407 in MDBK cells further supports its efficient in vitro replication capacity. Nevertheless, because viral strains, harvest times, passage backgrounds, and TCID_50_ assay conditions differ among studies, direct comparison of viral titers should be interpreted cautiously.

Previous BEV isolation and detection studies have shown that this virus can be found in cattle of different ages or production stages. In an early study, Cliver and Bohl isolated enteroviruses from rectal swabs of calves older than 4 weeks in dairy herds [27], Schiøtt and Hyldgaard‐Jensen [28] isolated the KO‐114 strain from a 6‐month‐old calf, and Blas‐Machado et al. [5] isolated BEV‐1 from a 2‐year‐old cow. Compared with these reports, the isolation of BEV NM2407 from the fecal sample of an 8‐month‐old diarrheic Holstein calf in the present study also suggests that BEV infection or shedding may not be restricted to young calves, but may occur in older calves and adult cattle at different stages. However, systematic comparisons of BEV infection rates, shedding levels, and clinical significance among cattle of different age groups are still lacking, and larger‐scale epidemiological investigations are needed for further clarification.

BEV‐related studies usually determine species assignment based on complete or near‐complete genome sequence analysis and perform subtype classification and phylogenetic verification mainly based on VP1, or in combination with P1 and polyprotein sequences [29]. In this study, BEV NM2407 was classified as the EV‐E4 subtype based on whole‐genome and VP1 phylogenetic analyses, with VP2 phylogeny used as additional support. This classification is consistent with the BEV genome reported by Luo et al. [30].

Because recombination events have been reported in BEV, 36 publicly available complete‐genome reference sequences were included together with the complete genome sequence of BEV NM2407 obtained in this study to construct a homologous recombination analysis dataset containing 37 sequences. Based on this dataset, RDP and SimPlot analyses identified two putative recombination breakpoints in the BEV NM2407 genome, located approximately at 1950 nt and 5850 nt. The 1–1950 nt region and the region from 5851 nt to the 3^′^ end showed higher local similarity to EV‐C‐related reference sequences in the dataset analyzed in this study, whereas the 1951–5850 nt region showed higher local similarity to EV‐D‐related reference sequences. These findings suggest that BEV NM2407 may contain potential recombination signals involving these genomic segments. Previous studies of BEV and related enteroviruses have also suggested that recombination plays an important role in viral evolution. Genetic diversity and recombination have been reported among BEV strains in China, suggesting that intra‐ or interspecies genetic exchange may occur during BEV circulation [4]. The recently reported recombinant BEV HeN‐2022 was considered to be related to BEV‐E1‐, BEV‐E4‐, and BEV‐F1‐related lineages, with putative recombination breakpoints involving the VP1 structural gene and the 5^′^UTR region [17]. Another study reported that ovine enterovirus 1 showed interspecies recombination characteristics involving bovine and porcine enteroviruses, with its 5^′^UTR showing high similarity to BEVs and the putative breakpoint located near the end of the 5^′^UTR [19]. Compared with these studies, the recombination signals identified in BEV NM2407 involved EV‐C and EV‐D sequences, suggesting that this isolate may have genomic mosaic features different from BEV‐E/BEV‐F‐related recombination events. It should be noted that the EV‐C‐ and EV‐D‐related sequences identified by RDP and SimPlot represent only the closest available matching sequences in the dataset analyzed in this study and should not be directly interpreted as definitive biological parental strains. Therefore, these results are more appropriately interpreted as evidence of genomic mosaic features and potential recombination signals in BEV NM2407, rather than as direct evidence for a clearly defined cross‐species recombination origin. Future studies incorporating more complete‐genome sequences of BEV and other animal‐derived enteroviruses are still needed to further verify the stability and evolutionary significance of this recombination signal.

VP1 sequence and structural analyses showed that BEV NM2407 retained a conserved VP1 core and the typical *β*‐barrel capsid scaffold in enteroviruses [31]. More obvious variation was observed in the surface‐exposed BC‐loop, DE‐loop, and GH‐loop regions, which are often involved in receptor engagement and neutralizing antibody recognition [11, 32]. Although homologous recombination analysis suggested that some genomic regions were more closely related to EV‐C‐/EV‐D‐related lineages, the VP1 amino acid composition and overall surface conformation remained more similar to the bovine EV‐E4 reference strain. This suggests that the capsid surface of BEV NM2407 remains broadly consistent with bovine EV‐E4‐like structural features, although direct evidence for altered antigenicity or changes in immune selection pressure was not obtained in this study.

To clarify the clinical manifestations, viral shedding, and tissue distribution of BEV NM2407 after inoculation in calves, a preliminary pilot experiment was performed before the formal animal experiment. Based on viral shedding and clinical observations from the pilot experiment, a relatively intensified inoculation design was used in the formal experiment to increase the likelihood of establishing consistent infection and observing tissue‐associated lesions under controlled conditions, rather than to fully mimic natural field exposure. The selection of 6 dpi as the necropsy time point was based on viral shedding and tissue viral RNA detection in the pilot experiment and was also informed by acute‐phase tissue sampling and necropsy time points used in calf infection models of BRV, BCoV, and BVDV.

In experimentally challenged calves, BEV RNA was detected in both nasal and rectal swabs, with rectal shedding generally occurring earlier and at higher levels than nasal shedding. A secondary increase in rectal viral RNA shedding was observed at 6 dpi, which may reflect continued or delayed intestinal viral replication and release into feces; however, intermittent fecal sampling, individual variation, and the limited observation period may also have contributed to this pattern. Because sampling ended at 6 dpi and tissues were collected only at this time point, the biological significance of this secondary increase remains uncertain. Relatively higher viral RNA loads were also detected in the trachea, lung, cecum, and rectum. These findings suggest that BEV NM2407 can establish infection in calves and shows certain respiratory and intestinal tissue distribution characteristics. Previous BEV infection studies have also shown variable clinicopathological outcomes. Blas‐Machado et al. [5] detected viral localization mainly in intestinal tissues and associated lymphoid tissues of experimentally inoculated calves, but no obvious clinical signs were observed. Ji et al. [16] reported that BEV‐F1 infection in ICR mice caused certain histopathological changes, although typical disease symptoms were absent. These studies suggest that BEV infection can establish replication and tissue distribution in vivo, but does not necessarily cause severe or consistent clinical disease.

In the present study, two challenged calves developed diarrhea, but the gross and histopathological changes were generally mild and localized. Gross findings were limited mainly to intestinal hyperemia/congestion, whereas histopathological changes mainly involved the jejunum, ileum, and rectum. Importantly, because viral antigen or viral RNA was not spatially demonstrated in tissues by an optimized IHC assay or in situ hybridization, the direct association between the observed histologic changes and BEV localization in affected tissues remains a limitation of this study. Future studies using commercial antibodies, optimized IHC conditions, or in situ hybridization assays may help to confirm tissue tropism and lesion association more directly. Overall, the present results suggest that BEV NM2407 can establish infection in calves and may be associated with mild, localized respiratory and intestinal involvement under the experimental conditions used here. However, its definitive pathogenic role under natural conditions still requires further validation through larger‐scale epidemiological investigations and more systematic animal infection studies.

In conclusion, this study isolated and identified a BEV strain, BEV NM2407, from Inner Mongolia. Based on whole‐genome, VP1, and VP2 phylogenetic analyses, this strain was classified as the EV‐E4 subtype and showed potential recombination features. BEV NM2407 was able to establish infection in Holstein calves and was associated with mild, self‐limiting clinical signs under the experimental conditions used in this study, with viral shedding through the respiratory and digestive tracts, detectable viral RNA in multiple tissues, and relatively localized histopathological changes. These findings provide new data supporting further studies on the genetic evolution, VP1 structural variation, tissue tropism, and infection characteristics of BEV in its natural host.

## Author Contributions

Lin Hou and Wei‐Guang Zhou conceived and designed the study. Lin Hou and Wei‐Guang Zhou devised the experimental methods. Jia‐Lei Zhang and Ya‐Xing Ban curated the data. Zhi‐Chao Li provided resources. Lin Hou, Jia‐Lei Zhang, Qi An, Yi‐Yang Zou, Zi‐Yuan Li, Qiang Du, Xuan An, Shuai Liu, and Zi‐Tong Wang performed the experiments. Lin Hou and Jia‐Lei Zhang prepared the original manuscript draft. Zhi‐Dan Zhang, Chun‐Xia Chai, and Ni‐Ni‐Gen Xi reviewed and edited the manuscript.

## Funding

This work was supported by the Major Science and Technology Project of the Inner Mongolia Autonomous Region (Grant 2021ZD0013) and the Innovation and Integrated Application of Prevention and Control Technologies for Major Diseases in Herbivorous Animals (Grant YLXKZX‐NND‐012).

## Disclosure

All authors reviewed and approved the final version of the manuscript.

## Ethics Statement

All animal procedures were conducted in accordance with relevant animal welfare guidelines and were approved by the Animal Ethics Committee of Inner Mongolia Agricultural University (Approval No. NND2022114). All calves were confirmed to be healthy and BEV‐negative prior to inoculation, monitored daily throughout the study, and euthanized at the experimental endpoint for tissue collection.

## Conflicts of Interest

The authors declare no conflicts of interest.

## Supporting Information

Additional supporting information can be found online in the Supporting Information section.

## Supporting information


**Supporting Information** Additional supporting information may be found in the online version of this article: Table S1: Primers and probes used in the RT‐PCR and qRT‐PCR assays. Table S2: Representative Cq values for BEV qRT‐PCR assay controls and seven clinical fecal samples. Table S3: Reference sequences and associated metadata used for phylogenetic and recombination analyses. Figure S1: Validation of the in‐house rabbit anti‐BEV‐VP2 polyclonal serum. Figure S2: Indirect ELISA screening of preinoculation calf sera.

## Data Availability

The viral genome sequence generated in this study has been deposited in GenBank under accession number PV032957.1. The data supporting the findings of this study are available from the corresponding author upon reasonable request.
