# Supplementary material for: Characterization and Evolutionary Analysis of Type E4 Bovine Enterovirus From Clinical Cattle With Diarrhea in Inner Mongolia, China
Source: Transbound Emerg Dis. 2026 Sep 18;2026:2973871. doi: 10.1155/tbed/2973871 (PMC13587357; doi:10.1155/tbed/2973871)
Supplement: Supplementary file 1 [file TBED-2026-2973871-s001.zip › Supplementary_Materials 2973871 Revision 7.docx]

Supplementary TABLE S1. Primers and probes used in the RT-PCR and qRT-PCR assays.

| **Targets** | **Sequences (5'-3')** | **Product size (bp)** |
| --- | --- | --- |
| BEV | F:TTTAAAACAGCCTGGGGGTTGTAC  R:CGGAGTACCGAAAGTAGTCTGTTC | 667 |
| BRV | F:GTACGATGTGGCTAAACGC  R:ATGCTGCTACTGCTGGTGT | 387 |
| BVDV | F:TAGCCATGCCCTTAGTAGGAC  R:ACTCCATGTGCCATGTACAGC | 288 |
| BCoV | F:CGAGTTGAACACCCAGAT  R:GAGACGGGCATCTACACT | 230 |
| E. *coli* STa | F:GAAACAACATGACGGGAGGT  R:GCACAGGCAGGATTACAACA | 229 |
| BEV qRT-PCR | F1:GGCCGTGAATGCTGCTAATC  R1:CGCGTTACGACGTAGCAACA  Probe:Cy5-AACCTCCGAGCGTGTGCGCRC-BHQ3 |  |

Primer/probe references are provided in the main text.

Supplementary Method S1

The primers and probes used in this study were adopted from previously published assays and were used under the manufacturer-recommended reaction conditions. No additional systematic optimization of reaction parameters was performed. Before clinical sample testing, assay performance under our experimental conditions was confirmed using a previously confirmed positive sample or standard template as a positive control, nuclease-free water as a no-template control, and a negative extraction control.

For BEV qRT-PCR, the primers and probe were adopted from a previously published TaqMan-probe assay, which reported the primer/probe sequences, plasmid copy-number calculation, and standard-curve performance for BEV quantification. In the present study, we did not perform a separate systematic re-optimization of the published assay parameters; instead, assay performance under our experimental conditions was confirmed using the controls described above.

Supplementary TABLE S2. Representative Cq values for BEV qRT-PCR assay controls and seven clinical fecal samples

| Sample type | Target | Result | Cq |
| --- | --- | --- | --- |
| Positive control | BEV | Positive | 15.14 |
| Clinical sample 1 | BEV | Positive | 21.37 |
| Clinical sample 2 | BEV | Positive | 20.38 |
| Clinical sample 3 | BEV | Positive | 21.05 |
| Clinical sample 4 | BEV | Positive | 17.89 |
| Clinical sample 5 | BEV | Positive | 15.63 |
| Clinical sample 6 | BEV | Positive | 17.71 |
| Clinical sample 7 | BEV | Positive | 15.43 |
| Negative extraction control | BEV | Negative | Undetermined |
| No-template control | BEV | Negative | Undetermined |

Supplementary TABLE S3. Reference sequences and associated metadata used for phylogenetic and recombination analyses.

| Strains | Collection | Submitted year | GenBank accession | Subtype |
| --- | --- | --- | --- | --- |
| High Point | USA | 2003 | AY421762.1 | EV-A4 |
| BrCr | USA | 1995 | U22521.1 | EV-A |
| CA67-10387 | USA | 2004 | AY843298.1 | EV-B |
| Human enterovirus B | USA | 2000 | NC001472.1 | EV-B |
| Human poliovirus 1 Mahoney | USA | 2000 | NC002058.3 | EV-C |
| Ca98-10615 | USA | 2006 | DQ995644.1 | EV-C |
| Enterovirus 70 | UK | 2004 | NC001430.1 | EV-D |
| Fermon | USA | 2003 | NC038308.1 | EV-D |
| VG-5-27 | UK | 1997 | D00214.1 | EV-E1 |
| PA12-24791 | USA | 2013 | KC667561.1 | EV-E1 |
| EV_NGR_2017 | Nigeria | 2018 | MH719217.1 | EV-E2 |
| PS 42 | USA | 2005 | DQ092792.1 | EV-E2 |
| D 14/3/96 | Germany | 2005 | DQ092786.1 | EV-E3 |
| JL-DH13 | China | 2019 | MN598020.1 | EV-E3 |
| BEV-GX1901 | China | 2019 | MN607030.1 | EV-E4 |
| SL305 | Australia | 1999 | AF123433.1 | EV-E4 |
| SA18334 | China | 2023 | OQ744533.1 | EV-E5 |
| MexKSU/5 | Mexico | 2015 | KU172420.1 | EV-E5 |
| BEV-261 | Germany | 2005 | DQ092770.1 | EV-F1 |
| IL/alpaca | USA | 2013 | KC748420.1 | EV-F1 |
| BEV/Egypt/2014 | Egypt | 2014 | KM667941.1 | EV-F2 |
| 3A | USA | 2003 | AY508697.1 | EV-F2 |
| PS87/Belfast | USA | 2005 | DQ092794.1 | EV-F3 |
| BEV Ho12 | Japan | 2016 | LC150008.1 | EV-F3 |
| BEV IS2 | Japan | 2016 | LC150010.1 | EV-F4 |
| W1 | New Zealand | 2003 | AY462106.1 | EV-F4 |
| M80/UK/1960 | UK | 2024 | PQ415717.1 | EV-F5 |
| T11f/UK/1960 | UK | 2024 | PQ415718.1 | EV-F6 |
| SWUN-AB001 | China | 2016 | KU955844.1 | EV-F7 |
| AN12 | Japan | 2015 | LC038188.1 | EV-F8 |
| swine/K23/2008/HUN | Hungary | 2010 | HQ702854.1 | EV-G |
| EVG 08/NC_USA/2015 | USA | 2017 | KY761948.1 | EV-G |
| Simian enterovirus A | USA | 1999 | NC003988.1 | EV-H |
| 1715 UWB | USA | 2007 | AF326759.2 | EV-H |
| 1631 | USA | 2000 | NC010415.1 | EV-J |
| N125 | USA | 2007 | AF414372.2 | EV-J |

Supplementary Methods S2 Generation and validation of the in-house rabbit anti-BEV-VP2 polyclonal serum.

The full-length VP2 coding sequence of BEV NM2407 was amplified based on the viral genome sequence and engineered with a primer-introduced C-terminal 6×His tag. The resulting construct was transformed into Escherichia coli BL21 (DE3) competent cells, and recombinant VP2 protein expression was induced with 1 mM isopropyl β-D-1-thiogalactopyranoside (IPTG) at 37 °C for 4 h. The His-tagged VP2 protein was purified by Ni-NTA affinity chromatography under denaturing conditions, followed by refolding via stepwise dialysis. The purified recombinant VP2 protein was then used as the immunogen for polyclonal antibody production.

New Zealand White rabbits were immunized with the purified recombinant VP2 protein according to a standard immunization protocol. Briefly, the primary immunization consisted of 200 μg of refolded protein emulsified 1:1 with Freund’s complete adjuvant and administered by multi-site intradermal injection. Two subsequent booster immunizations, each with 100 μg protein emulsified with Freund’s incomplete adjuvant, were performed at 2-week intervals. Pre-immune serum was collected before the first immunization and used as a negative serum control. Post-immunization serum was collected 1 week after the final booster immunization, aliquoted, and stored at -80 °C for subsequent validation and use.

1. Indirect ELISA analysis

The reactivity of the anti-BEV-VP2 polyclonal serum was evaluated by indirect ELISA. Briefly, 96-well plates were coated with 10 μg/mL purified recombinant VP2 protein overnight at 4 °C and blocked with Protein-Free Blocking Solution (Yeasen, Cat. No. PS108P) for 30 min at 37 °C. Serial two-fold dilutions of pre-immune and post-immunization sera, ranging from 1:1,000 to 1:128,000, were added to the plates and incubated for 1 h at 37 °C, followed by incubation with HRP-conjugated goat anti-rabbit IgG (Invitrogen, C31460100) for 30 min at 37 °C. The reaction was developed with TMB (Beyotime, P0210-500mL) substrate and stopped with ELISA Stop Solution (Solarbio, Cat. No. C1058), and absorbance at 450 nm was measured using a microplate reader. The assay was used to qualitatively evaluate antibody reactivity between pre-immune and post-immunization sera across serial dilutions.

2. Western blot validation

The specificity of the anti-BEV-VP2 polyclonal serum was evaluated by Western blotting using BEV NM2407-infected and mock-infected MDBK cell lysates. MDBK cells were infected with BEV NM2407, and cell lysates were collected at 18 h post-infection. Western blot analysis using the anti-BEV-VP2 serum detected a specific VP2 band in BEV-infected MDBK cells, whereas no corresponding signal was observed in mock-infected cells. GAPDH was used as a loading control. Western blotting was performed only to validate the specificity of the in-house anti-BEV-VP2 polyclonal serum, which was subsequently used for IFA in the main study.

Supplementary FIGURE S1. Validation of the in-house rabbit anti-BEV-VP2 polyclonal serum. (A) Indirect ELISA analysis of pre-immune and post-immunization rabbit sera against purified recombinant BEV-VP2 protein. Post-immunization serum showed stronger VP2-specific reactivity than pre-immune serum across serial dilutions. (B) Western blot validation using BEV NM2407-infected and mock-infected MDBK cell lysates. A specific VP2 band was detected in BEV-infected cells but not in mock-infected cells. GAPDH was used as a loading control.

Supplementary Methods S3 Indirect ELISA screening of pre-inoculation calf sera

Before experimental inoculation, serum samples from the six enrolled calves were screened for BEV-VP2-reactive antibodies by indirect ELISA. Ninety-six-well ELISA plates were coated with purified recombinant VP2 protein overnight at 4 °C, washed with PBST, and blocked with Protein-Free Blocking Solution. Calf serum samples were diluted and added to the wells, followed by incubation at 37 °C for 1 h. After washing, HRP-conjugated anti-bovine IgG was added and incubated at 37 °C for 30 min. The reaction was developed with TMB substrate and stopped with ELISA Stop Solution, and the absorbance at 450 nm was measured using a microplate reader.

Positive and negative control sera were included in parallel. The P/N value was calculated as the ratio of the OD_450_ value of each serum sample to that of the negative control. Samples with P/N ≥2.1 were considered ELISA-positive. All six pre-inoculation calf serum samples showed low OD_450_ values close to the negative control and markedly lower than the positive control, with P/N values below the positive cutoff, indicating that no obvious BEV-VP2-reactive antibody signal was detected before experimental inoculation.

Supplementary FIGURE S2. Indirect ELISA screening of pre-inoculation calf sera. Serum samples from the six calves used in the animal experiment were tested by indirect ELISA using recombinant BEV NM2407 VP2 protein as the coating antigen. Positive and negative control sera were included in parallel. All six pre-inoculation calf sera showed P/N values below the positive cutoff of 2.1, indicating no detectable BEV-VP2-reactive antibody signal before experimental inoculation.
